# How Practical Are Fiber Supercapacitors for Wearable Energy Storage Applications?

**DOI:** 10.3390/mi14061249

**Published:** 2023-06-14

**Authors:** Parya Teymoory, Jingzhou Zhao, Caiwei Shen

**Affiliations:** 1 Mechanical Engineering Department, University of Massachusetts Dartmouth, Dartmouth, MA 02747, USA; pteymoory@umassd.edu; 2 Mechanical Engineering Department, Western New England University, Springfield, MA 01119, USA; jingzhou.zhao@wne.edu

**Keywords:** fiber supercapacitors, flexible, energy storage, wearable electronics, smart textiles

## Abstract

Future wearable electronics and smart textiles face a major challenge in the development of energy storage devices that are high-performing while still being flexible, lightweight, and safe. Fiber supercapacitors are one of the most promising energy storage technologies for such applications due to their excellent electrochemical characteristics and mechanical flexibility. Over the past decade, researchers have put in tremendous effort and made significant progress on fiber supercapacitors. It is now the time to assess the outcomes to ensure that this kind of energy storage device will be practical for future wearable electronics and smart textiles. While the materials, fabrication methods, and energy storage performance of fiber supercapacitors have been summarized and evaluated in many previous publications, this review paper focuses on two practical questions: Are the reported devices providing sufficient energy and power densities to wearable electronics? Are the reported devices flexible and durable enough to be integrated into smart textiles? To answer the first question, we not only review the electrochemical performance of the reported fiber supercapacitors but also compare them to the power needs of a variety of commercial electronics. To answer the second question, we review the general approaches to assess the flexibility of wearable textiles and suggest standard methods to evaluate the mechanical flexibility and stability of fiber supercapacitors for future studies. Lastly, this article summarizes the challenges for the practical application of fiber supercapacitors and proposes possible solutions.

## 1. Introduction

In recent years, there has been a substantial increase in demand for flexible and wearable electronics for health monitoring, implantable devices, and consumer applications [1,2]. While smart watches, wristbands, glasses, and e-textiles composed of semi-rigid components have been on the market for years, it is still challenging to commercialize fully flexible electronics [3,4]. It is even more challenging to create smart textiles that integrate flexible electrical components without restricting the users’ physical activities [5]. Among all electrical components, energy storage devices are always critical as they are the major component to supply power. To be integrated into future flexible electronics, especially smart textiles, energy storage devices are required not only to be flexible and durable, but also to provide sufficient energy and power while being safe [6,7].

The power sources for existing commercial wearable electronics are all based on lithium-ion batteries, which offer relatively high energy density but are insufficient in terms of flexibility and safety [8]. Typical electrode materials for lithium-ion batteries are intrinsically rigid and brittle. The chemistry of such batteries is potentially explosive and involves toxic chemicals. Furthermore, the electrodes of lithium-ion batteries deform while being charged and discharged, making them more susceptible to deformation-caused damages [9]. An excellent alternative for commercial lithium-ion batteries is flexible supercapacitors, which have been extensively investigated for flexible and wearable energy storage applications, because they offer flexibility, durability, and safety that cannot be matched by battery systems [10,11,12]. Supercapacitors are intrinsically safer than batteries because they rely on static charge storage mechanisms or fast surface reactions instead of vigorous reactions in bulk electrodes in batteries. Some electrode materials for supercapacitors such as carbon nanotubes and graphene are intrinsically flexible. Combined with flexible polymer-based electrolytes, it is sufficient to create fully flexible, durable, and safe supercapacitors.

Many of the reported flexible supercapacitors are in the form of thin films, in which two electrode layers are sandwiching an electrolyte/separator layer. Such configuration cannot be smoothly integrated into textiles, and it also sacrifices the wearer’s comfort by blocking the airflow through the textiles [13,14,15]. To address these problems, fiber supercapacitors are created, which are used as yarns or threads that are ready to be woven or knitted into textiles [16,17,18,19,20]. As a result, fiber supercapacitors are one of the most promising solutions to the power sources for future smart textiles [16,21,22,23,24,25].

Despite the promises of fiber supercapacitors and tremendous progress in this field in the past years, some important practical questions have not been well answered so far, which makes it difficult to assess the practicality and potential of fiber supercapacitors. For example, how much energy density is required of a fiber supercapacitor to power a realistic wearable device? Are reported energy and power densities of fiber supercapacitors already sufficient or still insufficient for practical applications? How flexible are the reported fiber supercapacitors compared to wearable textiles? Do we have to sacrifice wearers’ comfort to integrate such devices into smart textiles? This mini-review paper aims at providing brief answers to these questions and suggesting future research directions for more practical fiber supercapacitors. In other words, the objective of this mini-review is to show the feasibility and potential of flexible fiber supercapacitors in real-world applications by providing a comprehensive evaluation of the practicality and potential associated with flexible fiber supercapacitors. To do this, we first review the types and advantages of fiber supercapacitors. Then we compare the energy and power densities of reported fiber supercapacitors with the power requirements of various smart and wearable electronics to evaluate how practical their energy storage performance is. Afterward, we estimate and compare the mechanical flexibility of reported fiber supercapacitors with that of wearable yarns and textiles to understand how flexible those devices really are. Finally, the prospects for using fiber supercapacitors as power sources for future smart textiles and the challenges that must be overcome in order to fully leverage their potential in this domain are discussed.

## 2. Why Fiber Supercapacitor?

**Figure 1:**
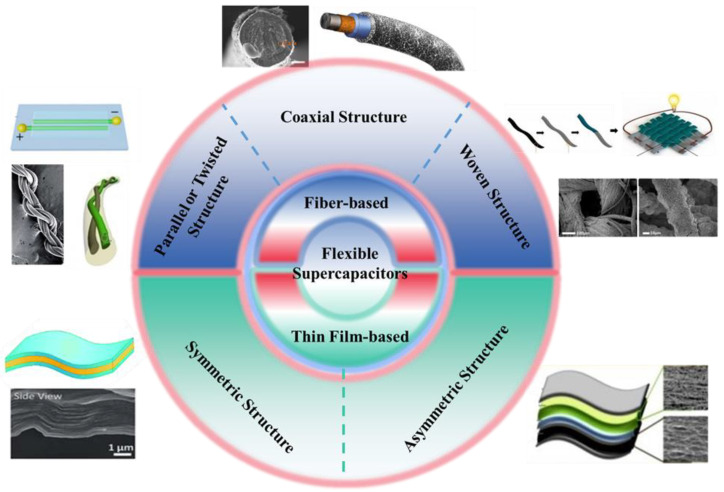
Types of flexible supercapacitors and their typical configurations. Images are adapted with permission from references [11,30,31,32,33,34,35].

Supercapacitors are energy storage devices with electrochemical properties that fill the gap between conventional capacitors and batteries [26]. Depending on the energy storage mechanism and active materials, supercapacitors can be classified into electric double-layer capacitors (EDLCs) and pseudocapacitors [27]. In EDLCs, the electrical charge is stored at the electrode/electrolyte interface by a physical process without Faradaic reactions. Typical electrodes for EDLCs are carbon-based materials with high specific surface areas, such as activated carbon, carbon nanotube, and graphene. The charge storage mechanism in pseudocapacitors, on the other hand, occurs through the fast and reversible surface redox process of active materials, such as transition metal oxides/hydroxides and conducting polymers. To make supercapacitors flexible, electrode materials are assembled in either fiber-based or thin film-based structures as shown in Figure 1. The flexibility of these supercapacitor devices is enhanced or limited by the intrinsic mechanical properties of electrode materials, electrolytes, substrates, separators, and the geometry and size of the overall structures [28,29].

Supercapacitors with thin film-based structures are usually assembled by sandwiching a layer of electrolyte-filled separator between two layers of electrodes. The two electrode layers can be made of the same material (symmetric type) or different materials (asymmetric type) [36]. Some active electrode materials are intrinsically flexible and free-standing, such as thin films assembled by carbon fibers, carbon nanotubes, and graphene [21,29,37,38,39]. However, these materials have high elastic modulus and limited flexibility unless the films are extremely thin. This leads to a significant trade-off between their flexibility and areal energy storage performance, in which thinner electrodes present higher flexibility but lower areal capacitance [40]. Other rigid and brittle electrode materials can also be assembled into flexible electrodes with the assistance of flexible substrates, such as plastic thin films and wearable textiles [21,29,37,38]. However, both the flexibility and energy density of such electrodes are limited by the substrates, which are electrically and electrochemically inert. Moreover, all of the thin film-based flexible supercapacitors will sacrifice the comfort of the wearers when integrated into wearable textiles, as they inevitably block airflow between the body and the environment [13].

The limitations of thin film-based supercapacitors for textile energy storage applications can be mitigated by utilizing fiber-based device configurations. Fiber-based flexible supercapacitors use coaxial, twisted, or parallel one-dimensional electrodes that integrate mechanical support, current collector, and active electrode materials in a single fiber/yarn/thread [14,16,41,42,43], with diameters ranging from micrometers to millimeters [44]. The fiber geometry with more degrees of freedom enables the highest possible flexibility of supercapacitor devices. Such designs also allow the devices to be integrated or directly woven into wearable fabrics without sacrificing the comfort of the wearers [45,46]. The excellent flexibility and small size can also make fiber supercapacitors more durable upon frequent mechanical deformations, such as bending, stretching, and twisting [47,48]. It is worth mentioning that fiber-shaped lithium-ion batteries have also been studied for the same purpose. Although such devices can achieve remarkable energy density (e.g., of 215 mWh/cm^3^ [49]), lithium-ion batteries have primary concerns regarding its safety and durability. Consequently, flexible fiber supercapacitors can be a better power solution for electronic and wearable devices, offering advantages in terms of safety and design [50,51].

Figure 1 summarizes the typical device configurations of flexible supercapacitors. This review paper only focuses on fiber-based supercapacitors, as they show significant advantages for textile energy storage applications over thin film-based ones. Three configuration designs are frequently reported for fiber supercapacitors. The first configuration composes of two parallel or twisted fiber electrodes with solid-state electrolytes in between [52,53]. Such configuration is relatively easy to fabricate and scale up as long as the two fiber electrodes can be continuously produced [34,54], but it may lead to high internal resistances if two electrodes are too far away from each other [33,55,56]. The second configuration consists of multiple layers of materials, including two electrodes and one layer of electrolyte, that are coaxially aligned into a core-shell structure within a single thread [30,57]. While this configuration is technically more challenging to manufacture, it can bring benefits such as more compact design, higher overall flexibility, and higher power density [58,59,60,61]. For practical application of the above fiber supercapacitors, threads or yarns with either parallel, twisted, or coaxial electrodes should be knitted or woven into textiles. Unlike the first two configurations in which both electrodes are aligned in the same direction and assembled in the same yarn, the third configuration of fiber supercapacitors has two electrodes coated with electrolytes in separate yarns. These yarns are then directly woven into supercapacitor textiles [32]. In other words, there is only one electrode per yarn in this case, and the supercapacitor is only functional when many yarns arranged in longitudinal and transverse directions are woven into a piece of fabric [32,62,63]. This design favors the manufacturing of large-area textiles but is challenging for small-scale integrations.

## 3. Electrochemical Performance

**Figure 2:**
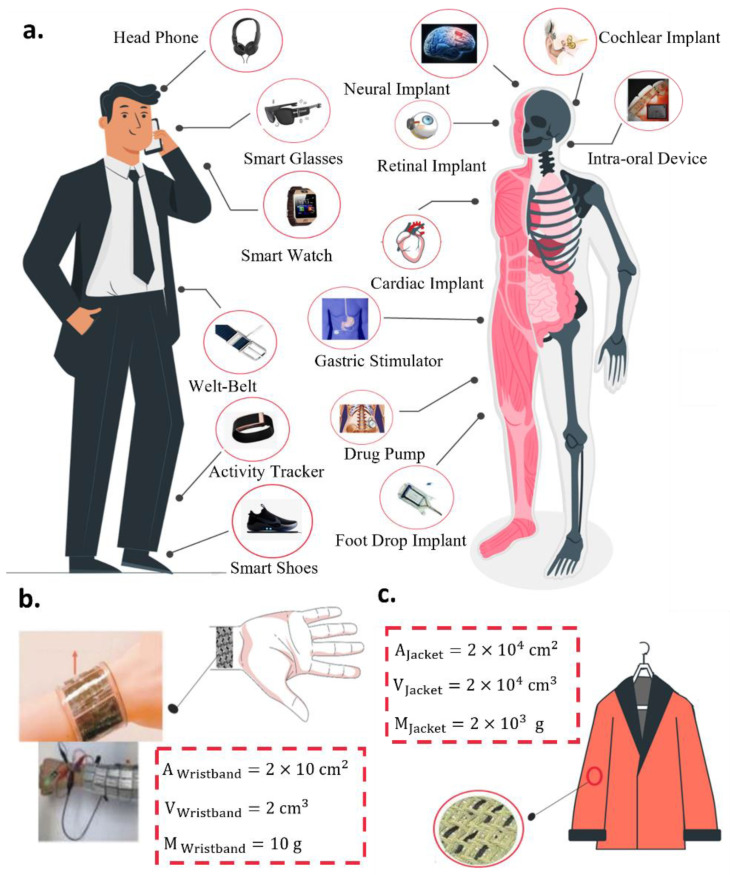
(**a**) Illustration of wearable and implantable electronics that can be powered by flexible supercapacitors made into (**b**) wristbands or (**c**) daily garments such as jackets.

Although supercapacitors offer many advantages over lithium-ion batteries, they still have a relatively lower energy density, i.e., lower energy storage capacity per unit volume. Flexible supercapacitors can have an even lower energy density than rigid ones when electrochemical performance has to be sacrificed for required flexibility. When used in wearable applications such as those illustrated in Figure 2a, however, the flexibility of the energy storage device has the additional advantage of being large in size without hindering other functions of the whole system. For example, the rigid battery that powers a smart watch has to be small to fit in the rigid body of the watch. Flexible supercapacitors, on the other hand, can be made into relatively large wristbands or straps [32] (Figure 2b). Even with lower energy density, a supercapacitor strap may store as much total energy as a small coin battery by utilizing more volume. This advantage can be fully exploited when our daily garments, such as the one illustrated in Figure 2c, are made of supercapacitor textiles. Our garments offer the greatest possible volume to store energy without putting an extra burden on our bodies. When this additional advantage is considered, we can assess the energy storage performance of fiber supercapacitors in a different way. We can assume that a large volume is available for those supercapacitors and compute the necessary energy and power densities to power practical wearable electronics.

**Figure 3:**
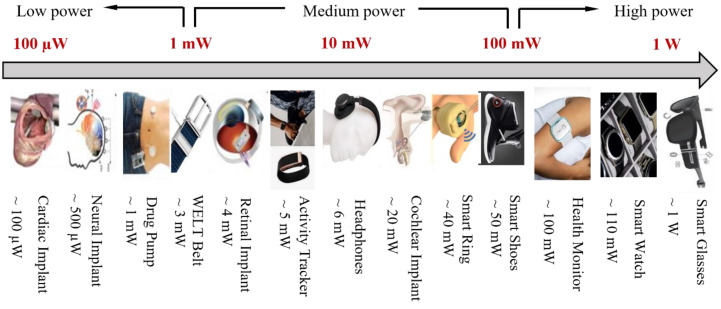
Typical average power consumption of wearable electronics [64,65,66,67,68,69,70].

To evaluate the practicality of the reported power or energy densities of fiber supercapacitors while assuming a large available volume, we need to know the power and energy consumption of practical wearable electronics. Figure 3 shows the power requirements for typical wearable electronics. Most of these devices consume power (denoted as P_Consume_) in the range of microwatts to hundreds of milliwatts. The power multiplied by 24 h would be the energy consumption per day for each of these devices. In most cases, we do not want to recharge these devices more than twice a day. So, we need the energy storage device that powers the corresponding wearable electronics to store at least the energy consumption per 12 h (denoted as E_Consume_). If we use a flexible fiber supercapacitor to power such a wearable device, we need it to supply power that is equal to P_Consume_ and provide energy of at least E_Consume_. If we assume the volume of this supercapacitor is V_SC_, we can compute the necessary power density (denoted as P′_SC_) and energy density (denoted as E′_SC_) of the supercapacitor using the equations below:

(1) $${P^{\prime }}_{SC}=\frac{P_{Consume}}{V_{SC}}$$

(2) $${E^{\prime }}_{SC}=\frac{E_{Consume}}{V_{SC}}=\frac{P_{Consume}\times 12\;h}{V_{SC}}$$

As discussed previously, the flexibility of fiber supercapacitor allows for using it to weave a wristband or even a piece of garment such as a jacket to power any electronics attached to it, instead of putting it inside the devices being powered. Therefore, we can assume that V_SC_ takes the value of at least the volume of a wristband (V_Wristband_), but probably not more than the volume of a jacket (V_Jacket_), as indicated by the equation below:

(3) $$V_{Wristband}\leq V_{SC}\leq V_{Jacket}$$

Combining Equations (1)–(3), we consider the power density (P′_SC_ in terms of mW/cm^3^) and energy density (E′_SC_ in terms of mWh/cm^3^) of a fiber supercapacitor to be practical if they satisfy Equations (4) and (5):

(4) $$\frac{P_{Consume}}{V_{Jacket}}\leq {P^{\prime }}_{SC}\leq \frac{P_{Consume}}{V_{Wristband}}$$

(5) $$\frac{E_{Consume}}{V_{Jacket}}\leq {E^{\prime }}_{SC}\leq \frac{E_{Consume}}{V_{Wristband}}$$

Equations (4) and (5) can also be interpreted as follows. If the power or energy density of a fiber supercapacitor reaches the upper limit in Equation (4) or (5), it can power the electronics after being made into a wristband. Or, if the power or energy density only reaches the lower limit, it will still be able to power wearable electronics after being woven into a jacket.

**Figure 4:**
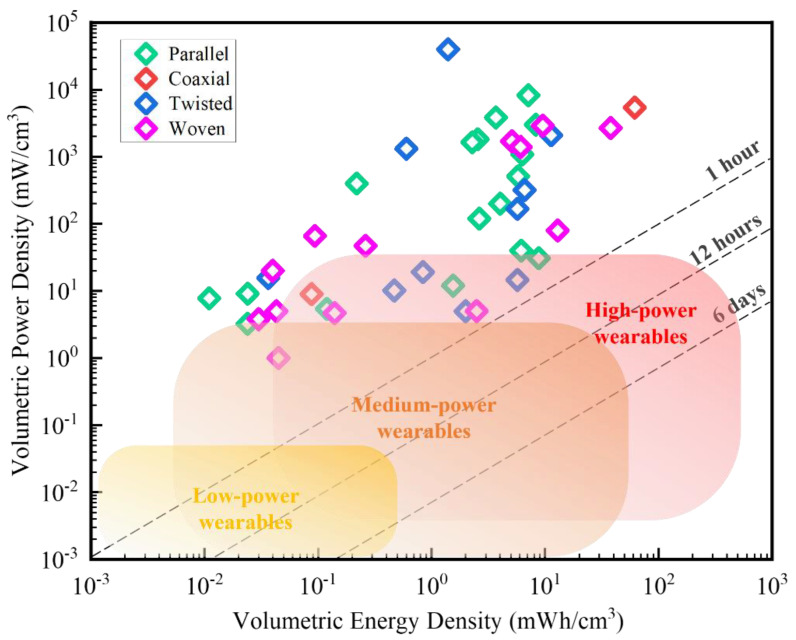
Ragone plot of reported energy and power densities of fiber-based flexible supercapacitors compared to the needs of typical wearable electronics.

In this review, the P_Consume_ value of each wearable device is extracted from Figure 3. The V_Wristband_ value is assumed to be about 2 cm^3^, which is the volume of a wristband that is 2 cm in width, 10 cm in circumference, and 0.1 cm in thickness. The V_Jacket_ value is assumed to be about 2 × 10^4^ cm^3^, which is the volume of a jacket that is 2 m^2^ in area and 1 cm in thickness. Based on these assumptions, we plot the required power density and energy density of fiber supercapacitors to power different types of wearable electronics in the Ragone plot in Figure 4. We can see that the power and energy requirements of wearable electronics fall into three categories: high-power, medium-power, and low-power.

Some wearables consume high power (over 100 mW on average) because they use relatively large screens, high computational power, and frequent wireless communication. To power these devices for 12 h, an energy capacity of over 1200 mWh is required. If we store such energy in a wristband supercapacitor with a small volume (2 cm^3^), we need a power density of 50 mW/cm^3^ and an energy density of 600 mWh/cm^3^. However, if we store the energy in a jacket supercapacitor with a higher volume (2 × 10^4^ cm^3^), we only need a power density of 0.005 mW/cm^3^ and an energy density of 0.06 mWh/cm^3^.

The power consumption of wearables falls below 100 mW if less screen lighting and on-site computation are needed. These medium-power wearables mainly use power for wireless communications. When wireless communications are not frequently used, some low-power wearables consume even less than 1 mW of power (Figure 3). The required power and energy densities for flexible supercapacitors to power those devices can also be estimated in the same way as mentioned above. The required ranges of power and energy densities needed to power typical high-power, medium-power, and low-power wearables are indicated as different blocks in the Ragone plot shown in Figure 4.

To assess the performance of fiber supercapacitors reported in the literature, we include representative energy and power densities of those devices in the same graph in Figure 4 (See details and references in Table 1). We can see that the configuration of the fiber supercapacitors (either parallel, twisted, coaxial, or woven configuration) does not have a major effect on the energy or power density of the devices. They usually show energy densities between 0.01 and 100 mWh/cm^3^ and power densities between 1 and 10^5^ mW/cm^3^. Compared with the needs of typical wearables, almost all reported fiber supercapacitors provide more than enough power densities. Many of them also provide energy densities that fall into the range of required values for high-power and medium-power wearables. Among the devices with the highest performance, quite a few show energy densities near 10 mWh/cm^3^. Those supercapacitors can power 100 mW wearables for 12 h if they are scaled up to a total volume of 120 cm^3^, which corresponds to the volume of a 40 cm-long, 30 cm-wide, and 1 mm-thick fabric.

Although the reported power and energy densities of many fiber supercapacitors seem sufficient to power a variety of wearable electronics, there are other practical factors that are not considered. One factor is the difference between the device volume and the textile volume. Many fiber supercapacitors normalize their energy by the volume of the fiber-shaped device. Some of them may only consider the volume of active electrodes excluding substrates, separators, current collectors, and protection/package layers. There will be considerable void space between functional fibers when they are woven into wearable textiles. Moreover, to ensure the flexibility and durability of the resulting textiles, looser weaving methods or additional coatings may be used. As a result, the true energy density normalized by the total volume of the textiles made from fiber supercapacitors can be more than one order of magnitude smaller than the reported values. Another factor is the difference between the lab-testing conditions and the practical usage. Practical applications require hours of continuous use of energy storage devices, resembling low-power discharging tests. However, almost all reports obtain their energy and power values through tests within 1 h. The energy density obtained through fast charging/discharging may not be fully used during slow discharging, especially for supercapacitors with significant self-discharging problems [71].

In summary, the reported fiber supercapacitors usually show sufficient power density for all kinds of wearable devices. Many of these supercapacitors seem to also provide sufficient energy density to power a variety of wearables for more than 12 h if they can be made into textiles with sufficient sizes. To make it easier to evaluate the practicality of fiber supercapacitors, future reports are recommended to report the volumetric performance normalized by the total size of textiles made by these fiber devices. It is also highly recommended to test the extractable energy at slow-discharging mode for at least 12 h or no shorter than one use cycle in practical applications.

**Table 1:** Electrochemical performance and durability of representative fiber-based supercapacitors.

Device Architecture	Volumetric Energy Density (mWh cm^−3^)	Volumetric Power Density (mW cm^−3^)	% Capacitance Retention after Bending	% Capacitance Retention after Cycle (Cycle Life)	Refs.
Parallel	8.3	3 × 10^3^	89.9% (1 K Bendings)	71.4% (10 K Cycle)	[33]
Parallel	7.13	8249	~97% (200 Bendings)	95% (10 K Cycle)	[72]
Parallel	24 × 10^−3^	3.25	99% (100 Bendings)	69% (10 K Cycle)	[53]
Parallel	8.8	30.77	-	86% (17 K Cycle)	[52]
Parallel	1.55	12	95% (20 Bendings)	-	[73]
Parallel	6.6	320	-	89% (5 K Cycle)	[74]
Parallel	2.63	120	-	88.1% (15 K Cycle)	[75]
Parallel	0.12	5.4	92% (3 K Bendings)	90.7% (5 K Cycle)	[76]
Parallel	4.02	200	-	90% (3 K Cycle)	[77]
Parallel	0.011	7.8	-	100% (1.4 K Cycle)	[56]
Parallel	24 × 10^−3^	9.07	-	100% (15 K Cycle)	[55]
Parallel	3.7	3840	-	98.5% (10 K Cycle)	[78]
Parallel	0.22	400	-	84% (10 K Cycle)	[79]
Parallel	6.16	40	-	86.7% (1 K Cycle)	[80]
Parallel	6.3	1085	~97% (1 K Bendings)	93% (10 K Cycle)	[81]
Parallel	2.55	1.82 × 10^3^	98% (1 K Bendings)	99% (1.6 K Cycle)	[82]
Parallel	2.29	1.64 × 10^3^	~100% (5 K Bendings)	71.9% (3 K Cycle)	[83]
Parallel	5.8	0.51 × 10^3^	-	-	[84]
Coaxial	0.088	9	96.8% (5 K Bendings)	88.4% (6 K Cycle)	[85]
Coaxial	84.2	73.8	99% (1 K Bendings)	82% (10 K Cycle)	[60]
Coaxial	11.8	0.1 × 10^3^	90% (1 K Bendings)	80% (1 K Cycle)	[61]
Coaxial	61.6	5.4 × 10^3^	-	85% (10 K Cycle)	[86]
Coaxial	3.84	0.018 × 10^3^	98% (200 Bendings)	~90% (2 K Cycle)	[87]
Twisted	0.47	10.18	80% (1 K Bendings)	-	[88]
Twisted	2	5	-	84% (5 K Cycle)	[89]
Twisted	11.3	2100	~100% (5 K Bendings)	85% (10 K Cycle)	[90]
Twisted	1.4	40 × 10^3^	98% (2 K Bendings)	94% (10 K Cycle)	[91]
Twisted	0.17 × 10^−3^	0.1	-	92% (500 Cycle)	[92]
Twisted	0.601	1320	-	-	[93]
Twisted	0.84	19.1	80% (1 K Bendings)	100% (1 K Cycle)	[54]
Twisted	3	0.55 × 10^3^	95% (1 K Bendings)	92% (10 K Cycle)	[94]
Twisted	36.4 × 10^−3^	15.6	-	90% (800 Cycle)	[95]
Twisted	0.22 × 10^−3^	1.32 × 10^3^	-	-	[96]
Twisted	6.6	320	~100% (2 K Bendings)	93% (5 K Cycle)	[34]
Twisted	5.7	14.5	~100% (5 K Bendings)	96.2% (5 K Cycle)	[97]
Twisted	5.7	167.7	-	~97% (5 K Cycle)	[97]
Woven	9.56	2.91 × 10^3^	~100% (1 K Bendings)	~100% (10 K Cycle)	[98]
Woven	0.263	47	-	85% (1 K Cycle)	[99]
Woven	0.14	4.68	~100% (1 K Bendings)	82% (1 K Cycle)	[62]
Woven	5.1	1700	-	~100% (20 K Cycle)	[100]
Woven	43 × 10^−3^	5	-	76% (3 K Cycle)	[101]
Woven	30 × 10^−3^	3.8	-	-	[101]
Woven	0.094	66.2	-	~105% (15 K Cycle)	[102]
Woven	3.6	-	~100% (1 K Bendings)	80% (10 K Cycle)	[32]
Woven	0.04	20	-	~97% (30 K Cycle)	[35]
Woven	37.8	2678	-	~98% (5 K Cycle)	[63]
Woven	0.045	1	95% (20 K Bendings)	97% (20 K Cycle)	[103]
Woven	2.5	5	96.8% (1 K Bendings)	90.4% (10 K Cycle)	[104]
Woven	12.9	80	97.1% (1 K Bendings)	-	[105]

## 4. Mechanical Performance

The analyses in the previous section have been based on the assumption that the reported fiber supercapacitors can be made into wearable textiles without affecting the wearers’ comfort. This assumption, however, only holds true when the mechanical flexibility and durability of such supercapacitor textiles are comparable to those of regular wearable textiles. Therefore, mechanical flexibility and durability, other than energy and power densities, are also essential factors that determine the practicality of fiber supercapacitors.

Most of the studies in the literature evaluate the flexibility of fiber supercapacitors by measuring their electrochemical performance under different bending angles (e.g., from 0 to 180 degrees) and their durability by checking the capacitance retention after various bending cycles (e.g., hundreds or thousands of bending cycles, as listed in Table 1). The durability tests are meaningful for proving practicality, as wearable textiles are constantly bent, twisted, stretched, and folded. The bending angle, however, does not reflect the true flexibility of a device. A film made of any material, even a piece of steel, can be bent to any angle as long as it is long enough and the bending radius is large enough. In this sense, the bending radius is a better indicator of flexibility than the bending angle. Some extreme deformations, such as folding, are actually equivalent to bending at an extreme radius. Many reports among others have successfully demonstrated high flexibility and durability through bending at extremely small radii [59] or folding for many cycles [40,106,107], which are recommended tests for future fiber supercapacitors.

**Figure 5:**
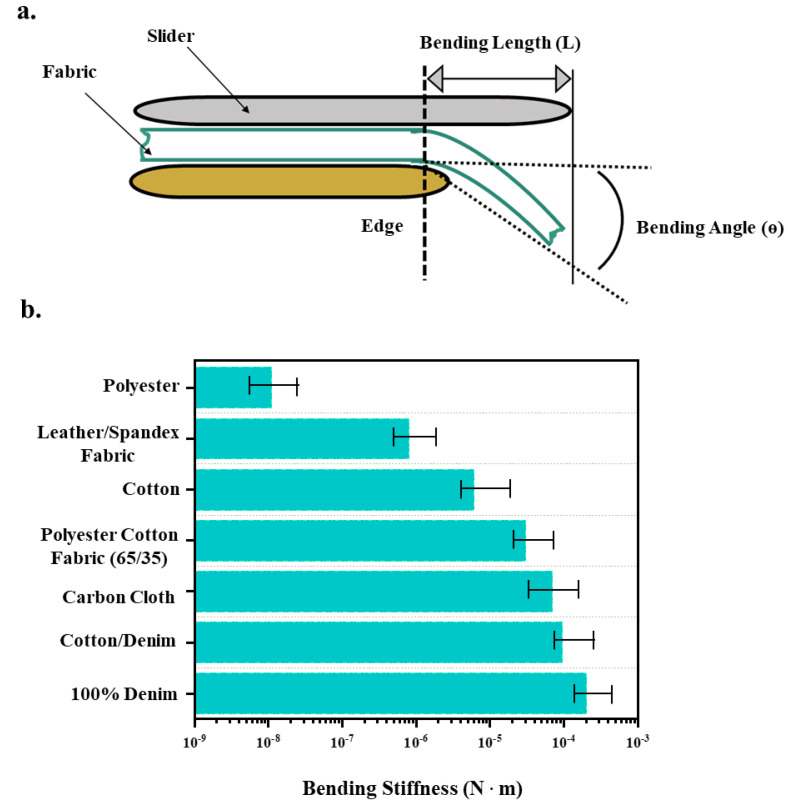
(**a**) Illustration of the bending stiffness test setup that is widely used in textile industry. (**b**) Typical bending stiffness values of commercial fabrics measured through the setup illustrated in (**a**) [110,111,112,113,114].

An even better way to evaluate the flexibility of supercapacitor textiles for wearable applications is to compare their bending stiffness or flexural rigidity with typical wearable textiles. In fact, the textile industry has developed standard protocols, such as Peirce’s Cantilever Test [108] and ASTM D1388 standard [109] (also illustrated in Figure 5a), to measure the bending stiffness of textiles to indicate their flexibility. The test without an applied load other than the self-weight is simple and effective and has been used extensively in the textile industry. It can also be used to assess the flexibility of supercapacitor textiles [40].

In such a test, a swatch of textiles is positioned under a weighted slider and moved over the edge of the fixture until the end of the cloth bends to a predetermined angle under its own weight (Figure 5a). The bending stiffness of the textile is calculated based on the traditional Peirce equation as follows:

(6) $$G_{Textile}=0.1\;{MC}^{3}$$

where G_Textile_ is the bending stiffness (N·m), M is the textile weight per unit area (N/m^2^) and C is the bending length (m). Here, a greater bending stiffness indicates a higher rigidity and lower flexibility. So, the flexibility of a textile or a supercapacitor textile is inversely related to its bending stiffness.

Figure 5b shows the bending stiffnesses of several commercial fabrics, most of which are evaluated at a thickness of 0.5 mm [110,111,112,113,114]. We can see that most of the wearable fabrics have bending stiffnesses between 10^−8^ and 10^−4^ N·m. To realize wearable supercapacitor textiles without affecting the wearers’ comfort, the bending stiffness of the supercapacitor textiles should also fall within this range.

Up to now, the bending stiffness of fiber supercapacitors is rarely reported. Even if the bending stiffness of a fiber-shaped device is known, it is still difficult to estimate the bending stiffness of the textile made by such fibers as there are different ways to weave or knit the fibers into textiles. Nevertheless, it is valuable to discuss the possibility to achieve reasonable bending stiffness for supercapacitor textiles. Theoretically, the bending stiffness of a rectangular thin film ($G_{Film}$) is proportional to the material’s elastic modulus (E) and the cube of film thickness (h) as shown in Equation (7) [115].

(7) $$G_{Film}\propto E\;h^{3}/12$$

The equation tells us that a desired bending stiffness or flexibility of a piece of textile or fabric can be achieved through the design of the material’s elastic modulus and the control of its thickness. In fact, most of the materials for flexible supercapacitors can be considered composites that combine electrode materials and polymer-based electrolytes [40]. Although some frequently reported electrode materials such as carbon nanotubes and graphene are considered intrinsically flexible materials, they actually have extremely high elastic modulus ranging from 100 to 1000 GPa [39,116]. They are only flexible when made into very thin films or incorporated in flexible matrix materials with low volume fractions. Almost all other high-performance electrode materials have similar issues. This results in a significant trade-off between the flexibility and energy density of flexible supercapacitors. To improve the flexibility, we should reduce the volume fraction of the high-modulus electrode materials in the electrode-electrolyte composite that constitutes the supercapacitor device, which will inevitably reduce the total energy that can be stored per device volume. However, such a strategy has seldom been considered as previous studies emphasize more on the energy density of individual supercapacitor devices. Besides the adjustment of elastic modulus of single fiber supercapacitors, another way to make supercapacitor textiles more flexible is to weave or knit fiber devices loosely. This will also reduce the potential energy density of the resulting textile, but the effects have not been quantified and are worthy of consideration.

In summary, it is difficult to evaluate the flexibility of existing fiber supercapacitors for practical applications as most of them only report flexibility in terms of being able to bend to certain angles. It is recommended that future studies evaluate the flexibility of textiles made from fiber devices in terms of bending radius, folding cycles, and bending stiffness measured in the same way as wearable textiles. The trade-off between the flexibility and energy density of supercapacitor textiles is also worth studying, as certain flexibility is needed to realize their full potential for practical applications discussed in the previous section.

## 5. Summary and Outlook

Fiber supercapacitors are promising energy storage technologies for future wearable electronics and have been intensively studied over the past decade. It is now the time to assess the practicality of such devices.

In terms of electrochemical performance, it is not surprising that the reported fiber supercapacitors usually show sufficient power density for all kinds of wearable electronics. The promising news is that many of them can also provide sufficient energy to power a variety of wearables if they were made into large pieces of wearable textiles. Future reports of fiber supercapacitors are recommended to measure the true available energy at a slow-discharging mode for at least the period of one use cycle in practical applications. It is also more meaningful to report the energy density normalized by the total size of textiles made from fiber-based devices instead of the size of individual fibers.

In terms of mechanical performance, the flexibility and durability of existing fiber supercapacitors are difficult to evaluate when only the bending angle is considered. Future studies are recommended to report bending radius, folding cycles, and bending stiffness measured in standard ways as commercial wearable textiles. There should also be more studies on the trade-off between the flexibility and energy density of supercapacitor textiles.

To move forward, future studies should focus more on electrode or electrolyte material systems with higher energy density and higher flexibility without fearing moderate sacrifice in power density. We should also explore new electrode-electrolyte composite designs and different methods of integrating fiber supercapacitors into textiles to balance flexibility and energy density.

In addition to the above considerations, the development of flexible fiber supercapacitors also presents various challenges related to manufacturing, integration, and long-term stability.

1. Scalable Manufacturing: Ensuring scalability and cost-effectiveness in manufacturing processes is challenging. Current methods are often intricate and time-consuming, hindering scalability. Identifying efficient techniques that can be seamlessly incorporated into large-scale production is vital for commercial viability.
2. Integration and Connectivity: The integration and connectivity of individual fibers within the supercapacitor structure pose critical challenges. Arranging and interconnecting the fibers to maximize electrode area and minimize electrical resistance is imperative. Maintaining consistent and reliable electrical connections between fibers is vital for sustained device performance.
3. Long-Term Stability: Ensuring the long-term stability and reliability of flexible fiber supercapacitors in diverse environmental conditions is another challenge. Temperature, humidity, and chemical exposure can impact device performance and lifespan. Developing protective coatings or encapsulation strategies is necessary to enhance environmental stability.

Addressing these challenges requires interdisciplinary research and collaboration among materials scientists, electrochemists, engineers, and manufacturing experts. Overcoming these obstacles will contribute to advancing and widely adopting flexible fiber supercapacitors in applications such as wearable electronics, energy storage devices, and smart textiles.

## Data Availability

The data presented in this study are available within this review paper.
